# The challenge of admitting the very elderly to intensive care

**DOI:** 10.1186/2110-5820-1-29

**Published:** 2011-08-01

**Authors:** Yên-Lan Nguyen, Derek C Angus, Ariane Boumendil, Bertrand Guidet

**Affiliations:** 1 Centre d'Epidémiologie Clinique, Hôpital Hôtel-Dieu, Assistance Publique des Hôpitaux de Paris, Paris, France; 2 Service de Réanimation Médicale, Hôpital Saint-Antoine, Assistance Publique des Hôpitaux de Paris, Paris, France; 3 Université Paris XI, Paris, France; 4 The CRISMA (Clinical Research, Investigation, and Systems Modeling of Acute Illness) Laboratory, Department of Critical Care Medicine, University of Pittsburgh Medical Center, Pittsburgh, PA, USA; 5 Department of Health Policy and Management, Graduate School of Public Health, University of Pittsburgh, Pittsburgh, PA, USA; 6 Unité 707, INSERM, Paris, France; 7 Université Pierre et Marie Curie Paris VI, Paris, France

## Abstract

The aging of the population has increased the demand for healthcare resources. The number of patients aged 80 years and older admitted to the intensive care unit (ICU) increased during the past decade, as has the intensity of care for such patients. Yet, many physicians remain reluctant to admit the oldest, arguing a "squandering" of societal resources, that ICU care could be deleterious, or that ICU care may not actually be what the patient or family wants in this instance. Other ICU physicians are strong advocates for admission of a selected elderly population. These discrepant opinions may partly be explained by the current lack of validated criteria to select accurately the patients (of any age) who will benefit most from ICU hospitalization. This review describes the epidemiology of the elderly aged 80 years and older admitted in the ICU, their long-term outcomes, and to discuss some of the solutions to cope with the burden of an aging population receiving acute care hospitalization.

## Epidemiology

### The aging of the world's population

Current forecasts predict that by 2050, the percentage of the population older than aged 80 years will double (Table 1). By 2050, people aged 80 years and older will represent 9.6% of the population in Europe (66,147,000 persons), 9% (35,813,000 persons) in North America, 6.5% (3,354,000 persons) in Oceania, 5.5% (40,098,000 persons) in Latin America and Caribbean, 4.4% (227,916,000 persons) in Asia, and 1.1% (21,336,000 persons) in Africa [1]. These population trends will lead to an increasing demand for healthcare resources (both in terms of number of beds and number of healthcare workers), including intensive care.

**Table 1:** Demographic projections*

Population > 80 yr, % (thousands)	2010	2020	2030	2040	2050
**Northern Europe**	4.5 (4,488)	5 (5,197)	6.4 (7,046)	7.5 (8,377)	8.9 (10,192)
**Southern Europe**	4.9 (7,641)	6.1 (9,676)	7.1 (11,259)	8.8 (13,872)	11.4 (17,759)
**Eastern Europe**	3.1 (9,246)	3.9 (11,304)	4.1 (11,543)	6.2 (16,526)	6.5 (16,762)
**Western Europe**	5 (9,536)	6.4 (12,261)	7.5 (14,483)	9.4 (18,292)	11.6 (22,366)
**Northern America**	3.8 (13,158)	3.9 (14,611)	5.3 (21,242)	7.3 (30,937)	8 (35,911)
**Central America**	1.3 (1,966)	1.6 (2,893)	2.2 (4,181)	3.3 (6,929)	4.8 (10,447)
**South America**	1.5 (5,810)	2.0 (8,495)	2.7 (12,632)	4.1 (19,809)	5.7 (28,005)
**Asia**	1.1 (47,200)	1.5 (69,800)	2.0 (99,786)	3.1 (158,863)	4.5 (232,127)
**Oceania**	2.8 (1,038)	3.1 (1,293)	4.2 (1,964)	5.3 (2,745)	6.3 (3,456)
**Africa**	0.4 (4,397)	0.5 (6,558)	0.6 (9,860)	0.8 (15,327)	1 (22,468)

*Source: Population Division of the Department of Economic and Social Affairs of the United Nations Secretariat, *World Population Prospects: The 2010 Revision*, http://esa.un.org/unpd/wpp/index.htm

Thus, if we maintain our current admission policy, intensive care resources must be expanded rapidly or will be quickly overwhelmed [2]. Bagshaw et al. predicted that by 2015 the rate of elderly aged 80 years and older admitted to the intensive care unit (ICU) will increase by 72%, representing roughly 1 in 4 admissions to the ICU [3]. Although there is variation in the current supply of critical care services across industrialized countries, these proportional changes are likely to be seen widely [4]. Given constrained healthcare financing and uncertainty regarding the benefits of critical care in some instances, simply increasing the quantity of critical care services is an unattractive policy. Instead, a more practical approach would be to try to define the most accurate criteria for identification of those likely to benefit from ICU care regardless of age.

### Aging of patients admitted in the ICU

There is currently an increasing demand for critical care resources, which may be explained by both underlying demographic changes and the growing prevalence of conditions that require intensive care management, such as severe sepsis or high-risk surgery [5]. During the past two decades, the number of elderly admitted to the ICU has increased. In a single-center Dutch study, Blot et al. found that the number of patients aged 75 years and older increased by 33% between 1992-1996 and 2002-2006 [6]. In a large multicenter cohort study that gathered the data of 57 ICUs across New Zealand and Australia (ANZICS CORE cohort), Bagshaw et al. reported an increasing number of admissions of elderly patients aged 80 years and older of roughly 6% per year between 2000-2005 [3]. In this cohort, the rate of admission of elderly aged 80 years and older represented approximately 14% of total admissions in 2005 [3].

The majority of the epidemiological studies of the elderly admitted to the ICU are single-center, which may result in selection bias, limiting our ability to understand the broad picture of the number of elderly aged 80 years and older currently admitted to the ICUs, the main diagnosis at admission, the amount of resources used, and patient-centered outcomes. To our knowledge, there also is no study published concerning socioeconomic status or race differences among the elderly admitted to the ICU. Such differences might be present despite the presence of a national healthcare system [7].

### Differences in care between old versus young patients

In a recent observational French study conducted in 15 emergency departments between 2004 and 2006, Garrouste-Orgeas et al. found that despite the existence of criteria indicating that ICU admission was appropriate, only 40% patients aged 80 years and older were referred to the ICU by the emergency physician and only half of them were finally admitted by the ICU physician [8].

There also are discrepancies between the young and old in terms of delay of treatments and use of recommended guidelines. In a large review of elderly older than aged 65 years suffering from acute myocardial infarction, Nguyen et al. reported that the elderly were more likely to have a longer prehospital delay than younger patients [9]. In a multicenter Swiss cohort, Shoenenberger et al. reported that, even after exclusion of patients with potential nonindications and adjustment for confounding factors (such as comorbidities), elderly patients aged 80 years and older with acute myocardial infarction were less likely to receive the recommended medical care (acetylsalicylic acid, clopidogrel, beta-blockers) and interventional care (thrombolysis and percutaneous coronary intervention) [10]. After being admitted to the ICU, there also are differences between young and old in terms of the intensity of treatment provided (e.g., vasopressor infusion, mechanical ventilation, and renal replacement therapy). Recent data suggest that the intensity of treatment for patients aged 80 years and older is increasing [11].

As with younger patients, men appear to be admitted more frequently than women among the elderly [12,13]. In a large multicenter Canadian cohort, Fowler et al. showed that despite a larger number of women being hospitalized, women aged 80 years and older with same admission type and severity of illness than men, were less likely to be admitted in the ICU and to receive mechanical ventilation [12]. Unfortunately, this study did not include any data on patient and family preferences, which might be gender-related.

## Patient-centered outcomes of elderly aged 80 years and older admitted to the ICU

During the past 20 years, the main primary outcome used in epidemiological studies of elderly patients admitted to the ICU was ICU or hospital mortality. However, as pointed out by the World Health Organization, health is not a matter of "the absence of disease or infirmity" but "a state of complete physical, mental and social well-being" [14]. Thus, the rationale for admitting an elderly patient to the ICU should not be restricted to short-term management of an acute disease but rather to allow her to recover from acute illness with a satisfactory quality of life. To describe patient-centered outcomes, we should consider two types of elderly admissions: planned surgical and unplanned surgical or medical admissions.

### Planned surgical admissions

Current studies suggest that elderly aged 80 and older hospitalized in the ICU after planned surgery have reasonable long-term outcomes. In a large multicenter cohort study of 120,123 admissions across 57 ICUs from the Australian New Zealand Intensive Care Society Adult Database (ANZICS), Bagshaw et al. found that the main reason for critical care admission of elderly patients aged 80 years and older was planned surgery [3]. ICU and hospital mortality were respectively 12% and 25%. Also, among survivors, 72% were discharged to home. In a Dutch single-center cohort study, de Rooij found that at 1 year, 57% of patients who had planned surgery survived and three-quarters of patients living at home before ICU admission were still living at home [15]. Also, they showed that nearly 90% of the survivors experienced mild or no cognitive impairment. However, the self-reported quality of life at follow-up (1 to 6 years after admission to ICU) was significantly lower than in the general population (68.4 ± 15.1 versus 72.5 ± 18.2), but it is possible that the patients who underwent surgery in the first place had a worse baseline quality of life than population controls. Data on patient and informal caregiver satisfaction on ICU admission are lacking.

### Medical and unplanned surgical admissions

Current studies suggest that elderly patients admitted for medical and unplanned surgical reasons have rather poor outcomes compared with those admitted for planned surgical admissions. Three single-center French cohort studies admitting predominantly elderly patients with medical conditions showed very high ICU (from 38% to 64%) and hospital mortality rates (from 45% to 55%) [11,16,17]. In the cohort of Tabah et al., 1-year mortality was 80% in the subgroup of medical patients and 67% in the subgroup of unscheduled surgery [16]. These results are consistent with the results of de Rooij et al. who reported a 1-year mortality rate of 89% for both medical and unplanned surgical admissions [15]. At 2 years after hospital discharge, Roch et al. estimated that the standardized mortality ratio was 2.56 (2.08-3.12) compared with the age- and gender-adjusted mortality of general population [17]. The ICE-CUB1 study focused on elderly patients (older than 80 years) visiting the emergency department of 15 different hospital located in Paris and suburb area [8]. All included patients had at least one condition that potentially required ICU admission. The triage process was drastic, because only one of eight patients was ultimately admitted to the ICU. The hospital and 6-month outcome of the entire cohort are depicted in Table 2. The independent factors for 6-month mortality are presented in Table 3. Among the 1,230 ICE-CUB1 patients who were alive 6 months after their emergency department visit, 1,085 had their functional status evaluated: 33.7% were independent for all activities listed in Katz's scale and 16.2% were unable to perform at least one activity that they had been able to perform at the time of the emergency department visit; 12% of ICU admitted patients experienced a minimum of one point loss in at least one dimension of the activities of daily living with respect to baseline during the 6 months after the emergency department visit. The proportion was similar in not admitted patients. Accordingly, in both groups, 6 months after the emergency department visit, 63% of patients had died or experienced functional deterioration [18].

**Table 2:** Mortality and functional status 6 months after visiting the emergency department

ICE-CUB1 study	Emergency triage		ICU triage		
	Too well	Too sick	Too well	Too sick	Admitted to the ICU
**Patients, N**	1339	642	155	170	316
**Hospital mortality (%)**	8	55	17	68	33
**6-months mortality (%)**	28	80	41	87	48
Decrease in ADL score*	0.62	0.52	0.01	0.41	0.44

*ADL = activity from daily living assessing functional status (scale from 0 (total limitation) to 6 (no limitation in all 6 activities)

**Table 3:** Independent factors for 6-month mortality: multivariate analysis of the ICE-CUB1 study

	In-hospital death	Death at 6 months
**Age (grand mean centered) per year**		1.04 (1.02-1.06)
**ADL per point**	0.79 (0.75-0.84)	0.85 (0.8-0.91)
**Demented (yes vs. no)**	0.61 (0.44-0.85)	
**Cancer** **(yes vs. no)**		2.59 (1.74-3.9)
**Normal appearance vs. emaciated**		0.82 (0.54-1.24)
**Somewhat malnourished appearance vs. emaciated**		0.48 (0.33-0.7)
**Decubitus ulcer** **(yes vs. no)**	1.53 (0.97-2.26)	

Only three recent studies focused on long-term follow-up for quality of life after ICU hospitalization [15-17]. These studies have small sample sizes due to the high 1-year mortality rates in these categories of patients, and this selection bias may induce discrepant results. For example, Tabah et al. found that at 1 year, quality of life was similar to that of the general population, whereas de Rooij et al. and Roch et al. found that quality of life was significantly lower (in terms of usual activities or physical components) [15-17]. Tabah et al. found that at 1 year, 80% were self-sufficient for activities of daily living, whereas de Rooij et al. found that respectively 53% and 73% of the patients surviving at 1 year after unplanned surgery and medical admissions suffer from four or more functional disabilities (modified Katz ADL index score) [15,16]. However, the cognitive status was relatively good at 1 year with respectively 63% and 75% with mild or no cognitive impairment in the Dutch cohort [15]. These poor outcomes in terms of physical and neuropsychiatric disabilities and impaired quality of life are consistent with data on long-term outcomes of intensive care survivors [19-23]. Barnato et al. showed that critically ill patients undergoing mechanical ventilation (mean age 76 ± 7 years) are more likely to suffer from greater disability compared with an age- and gender-matched population who incur hospitalization or not [19]. Unroe et al. reported that at 1 year, average critically ill patients receiving prolonged mechanical ventilation (mean age 55 ± 16 years) spent 74% of all days alive in a hospital postcare facility or receiving home health care [22]. Wunsch et al. showed that Medicare beneficiaries who survive intensive care (mean age 78 ± 7 years) had higher 3-year mortality than hospital controls [23]. Those who received mechanical ventilation or were discharged to a skilled care facility had an increased risk of death during the first 6 months after ICU hospitalization. Cuthbertson et al. found that 5 years after hospital discharge, cumulative quality-adjusted life-years was significantly lower in ICU survivors compared with the general population [21]. Desai et al. reported that survivors of critical illness are more likely to experience long-term physical, neuropsychiatric, and quality of life impairments [20].

Nevertheless, the external validity of the studies presented to other healthcare systems may be weak due to the differences of ICU organization and management between countries. Indeed, a recent study revealed large differences in case mix between patients admitted to U.K. versus U.S. ICUs [24].

## Strategies to cope with the burden of elderly patients who require acute care hospitalizations

As discussed earlier, elderly patients admitted to the ICU after planned surgery have reasonable long-term outcomes. On the other hand, long-term outcomes after ICU admission for unplanned surgical and medical elderly patients are rather poor. For this group, there are two broad options:

- Not admitting them to the ICU and privileging a hospitalization in a regular ward or acute care elderly unit.

- Admitting them to the ICU and conducting efforts to ensure a rapid ICU discharge.

### Not admitting

Triage decision is one of the hardest tasks of any intensivist. Part of the difficulty is accounting for evaluation of the severity of illness, the potential benefit of being hospitalized in the ICU, and beds availability in an emergency context [25]. Among the reasons for not admitting a patient to the ICU are: patient or family wishes for not escalating care, the futility of higher-level care (patient does not actually require intensive care, there are no expected benefits from critical care treatment or end-of-life planning). Moreover although ICU "often" is considered a safe environment by patient and family members, there are several risks associated with unnecessary intensive care (often neglected) that may delay or impede full recovery. Among the inherent risks, there is a greater exposure to nosocomial infections, iatrogenic complications from invasive monitoring, imposed bed/chair rest, sleep deprivation, delirium, increased hospital length of stay, and more restrictive visiting hours for families [26,27]. All of these risks may lead to increased morbidity, cognitive impairment, and functional disability [28,29].

As seen previously, expected benefits of medical or unplanned surgical ICU admissions of elderly patients aged 80 years and older are particularly weak and make ICU admission of these categories of patients questionable. To date, there is no randomized, controlled study available; the only available data came from observational studies with inherent limitations (retrospective collection of data at baseline, lack of a control group). Boumendil et al. recently reported in a multicenter observational study (including a majority of medical admissions) that ICU admission compared with admission to a regular ward did not improve the long-term survival of patients aged 80 years and older [30]. These results emphasized previous data of Martínez-Sellés et al. who reported that the outcome of persons aged 90 years and older admitted with acute myocardial infarction was not influenced by an admission to a coronary care unit [31].

An alternative to ICU hospitalization is admission to an acute care elderly unit. Current data suggest that elderly patients who are hospitalized for an acute medical illness suffer a functional decline afterwards [32]. Maximizing recovery of daily life activities may allow the elderly to be discharged home and to limit the burden for caregivers. Acute care units for the elderly were created during the early 1990s and initially included four components: a prepared environment, patient-centered care, medical care review, and planning for discharge [33]. A prepared environment is an ergonomic environment planned to limit risk of falls (e.g., uncluttered hallways and elevated toilet seats) and disorientation (e.g., using large clocks and calendar). Patient-centered care includes the daily assessment of physical, cognitive, and psychosocial function, protocols to improve self-care, continence, nutrition, mobility, sleep, skin care, mood, cognition, and daily rounds by a multidisciplinary team. A medical care review is a review of daily planned medicine and procedures and the use of protocols to minimize adverse effects. A planning for discharge is an early plan to facilitate home return and involve social workers. When posthospital care is needed, options may be large and the choice of a structure should depend on the patient's clinical status and care goals, family circumstances, and resources [34].

A recent review conducted by Ahmed et al. showed that acute care for the elderly units are associated with reduced functional decline, costs, hospital length of stay, and lower readmission rates to acute care hospitals compared with usual care [35]. The results of the prevalence and reduction of delirium were mixed. All surveys of patients, healthcare providers, and caregivers reported higher satisfaction for acute care for the elderly units.

### Admitting selected elderly aged 80 years and older to the ICU

With 1-year mortality rates of 80% or 90%, it seems reasonable that some portion of elderly patients may not be best served by ICU care. The difficulty is determining which subjects should not be admitted to the ICU. During the past decade, ICU admission criteria classically include severity of illness, comorbidities, the levels of frailty and disability, the expected impact of treatment on the outcome, the expression of wishes regarding do-not-resuscitate orders, and the availability of ICU beds [36]. Severity of illness was considered explaining "a small part of the increased hospital mortality" [36]. On the other hand, "functional status" was considered one of the major predictors of long-term outcome [36].

Recent data suggest that a greater age and a high level of severity of illness are predictive of poor outcomes. Sligl et al. reported in a multicenter British cohort study that among critically ill adult patients with pneumonia, age 80 years and older was an independent factor of death at 30 days (odds ratio (OR) = 2.54 [1.21-5.36]) as well at 1 year (3.47 [1.99-6.05]) [37]. Blot et al. showed in a Belgium single-center cohort study that among critically ill patients with nosocomial blood stream infections, age older than 75 years was associated with higher hospital mortality rates (OR = 1.8 [2.3-2.3]) [6]. Farfel et al. in a single-center Brazilian cohort study of elderly admitted to the ICU found that age 75 years and older was an independent risk factor of death but only for patients who required invasive mechanical ventilation (OR = 2.68 [1.58-4.56]) [38]. Concerning severity of illness, in a large cohort of American community elderly, Gill et al. reported that injuries and illnesses leading to hospitalizations are associated with increased disability and reduced recovery [39]. Iwashyna et al. in a national American cohort study of older patients with a mean age of 77 years demonstrated that severe sepsis is associated with cognitive impairment (moderate to severe cognitive impairment OR = 3.3 [1.5-7.25]) and functional disability (acquisition of 1.5 new functional limitation at hospitalization for severe sepsis) [40].

On the other hand, some data suggest the presence of comorbidities and functional status may be poor predictors of outcome. In a large American cohort of elderly patients, Yende et al. reported that prehospitalization comorbid conditions did not influence long-term mortality after pneumonia [41]. Barnato et al. reported in a cohort of elderly undergoing mechanical ventilation that prehospitalization functional status was not a good predictor of disability among survivors [19]. Similarly, Roch et al. found that preadmission functional scores of elderly aged 80 years and older before ICU admission, evaluated by the Knaus classification or the Karnofsky index, did not affect hospital or 2-year mortality [17].

Another challenge in the decision-making process of admission of elderly patients aged 80 years and older is that physicians' choices more often are intuitive than "rational." Overvaluing "impressions" and "intuitions" rather than using evidence-based decisions may lead to unintended consequences [42]. In a recent study, Rodríguez-Molinero et al. showed that the decision to admit an elderly patient to the ICU was essentially based on age and the physician's estimation of functional and mental status [43]. Unfortunately, the evaluation of functional and mental status of their patients by physicians was not concordant with evaluation by the family. For example, the functional status of patients rejected from ICU admission often was underestimated, whereas the functional status of patients admitted to the ICU often was overestimated.

Besides improving survival, one of the major goals of ICU admission for the elderly (and indeed all patients) is to avoid inherent risks and improve recovery. Then, efforts to ensure rapid discharge from the ICU (such as noninvasive care) should be promoted to limit a new or additional activity of daily living disability, which are associated with poor long-term outcomes [32].

## Conclusions

The aging of the population will lead to an increasing demand for critical care resources. Current data suggest that planned surgical patients aged 80 years and older may benefit from ICU care. However, for patients aged 80 years and older who are hospitalized for unplanned surgery or medical reasons, the benefits of an ICU hospitalization are unclear. For these patients, two options seem reasonable: 1) not admitting to the ICU but instead admitting to a regular ward or an acute care for elderly unit; or 2) admitting selected patients to the ICU and promoting efforts to ensure a rapid ICU discharge. Further studies are needed to evaluate the benefits of intensive care for this selection of patients.

## Competing interests

The authors declare that they have no competing interests.

## Authors' contributions

YLN, DCA, and BG contributed equally to the manuscript. AB read the final draft and added data on the study ICE CUB 1. BG is the primary investigator of ICE CUB1 and ICE CUB 2. AB is the méthodologist and statistician of ICE CUB 1 and ICE CUB 2.
